# Production of an eco-friendly concrete by including high-volume zeolitic supplementary cementitious materials and quicklime

**DOI:** 10.1038/s41598-023-50761-6

**Published:** 2024-01-02

**Authors:** Danutė Vaičiukynienė, Dalia Nizevičienė, Aras Kantautas, Gintautas Tamošaitis, Ignacio Villalón Fornés, Pavel Krivenko, Olha Boiko

**Affiliations:** 1 Building Materials and Structures Research Centre, Faculty of Civil Engineering and Architecture, Kaunas University of Technology, Studentu st. 48, 51367 Kaunas, Lithuania; 2 Department of Electrical Power Systems, Faculty of Electrical and Electronics Engineering, Kaunas University of Technology, Studentu st. 48, 51367 Kaunas, Lithuania; 3 Department of Road Engineering, Faculty of Engineering Sciences, Kaunas University of Applied Engineering Sciences, Tvirtovės al. 35, 50155 Kaunas, Lithuania; 4 Kyiv National University of Construction and Architecture (KNUCA), Kyiv, Ukraine

**Keywords:** Civil engineering, Materials science

## Abstract

The production of ordinary Portland cement (OPC) is one of the main global causes of CO_2_ release to the atmosphere. However, its availability and unique characteristics as a binding material make it difficult to be substituted by eco-friendlier materials. However, OPC partial replacement with pozzolanic materials is one of the best solutions to this problem. Hence, in this study, various types of high-volume zeolite were employed as supplementary cementitious materials (SCM), substituting the OPC by up to 50 wt.% in the composition of the created mortars. Besides, quicklime and inorganic accelerators were included in some of the mortar mixtures to improve the hydration reaction and enhance its speed. The mechanical, durability and durability in sea water properties were investigated. Although the usage of SCM caused a decrease in the mechanical and durability properties of the specimens, the addition of 10 wt.% quicklime palliated this degradation by enhancing by 40% the 28-days compressive strength of the specimens and by significantly improving their durability (porosity, freeze–thaw resistance and carbonation resistance). Moreover, the mixtures were proved to be resistance to aggressive ionic environments, since their compressive strength even increased after 28-day immersion in seawater, due to the additional formation of hydration compounds.

## Introduction

It is not a secret that sustainability is among the main global challenges of the current era and that, for this reason, scientific activity should move strongly towards sustainable development. One of the main areas which needs these kinds of solutions is the building industry, since it consumes humungous amounts of natural resources and contributes remarkably to the greenhouse effect. Hence, a new culture of sustainable building solutions is necessary. Sustainable buildings should be made from eco-friendly and carbon neutral materials, but this is not usually the case. In fact, the most widely used material in the building industry is the concrete, which has a gigantic carbon footprint, due to the emissions released to the atmosphere during the production of its main binder, the ordinary Portland cement (OPC).

A solution to this problem could be the use of supplementary cementitious materials (SCMs), which replace a certain amount of the concrete’s OPC binder, so reducing its carbon footprint. Usually, pozzolanic materials are those included as SCM in OPC systems, since they generally improve the long-term strength and the durability properties of concrete. For this purpose, some natural pozzolans can be used, such as clinoptilolite zeolite or volcanic tuff. However, aside from the natural resources, there is an increasing tendency to use pozzolanic industrial wastes as a means of developing circular economies. Some examples of this kind are fly ash, blast furnace slag, silica fume or synthetic zeolite, among others.

### Incorporation of high-volume SCMs to OPC systems

The more OPC is substituted by SCM, the eco-friendlier a building material is. In this sense, the incorporation of > 50 wt.% of SCM in a cementitious system is referred to as “high volume”. Numerous authors have investigated this kind of systems, although the effects of the high-volume substitutions in OPC systems have not been fully explored yet. El-Chabib et al.^1^ performed an investigation whereby high-volumes of SCM in binary, ternary or quaternary binder systems were included, achieving up to 70% cement replacement. In this way, concrete with similar properties to those of OPC-based concrete was produced. In other study, Ulusu et al.^2^ substituted clinker with high-volume pumice (up to 40%). It was observed that, with the enhancement of the pumice content, the compressive strength (*CS*) of mortars decreased, but, at the same time, their resistance to sulphates increased. The optimal amount of pumice was found to be 10 wt.% and 20 wt.%. Chindasiriphan et al.^3^ investigated concrete made with high volumes of ground and coarse bottom ash. The OPC and sand were substituted with two types of bottom ash: ground and coarse, respectively. After 90 days, the concrete with 65% of ground bottom ash exhibited a *CS* of 70 MPa. Besides, the incorporation of coarse bottom ash instead of fine aggregate led to the reduction of the autogenous shrinkage of concrete. Hence, this type of material can be classified as a sustainable high strength concrete. Wilson et al.^4^ substituted 60 wt.% of OPC by natural pozzolan and observed that the main characteristics of the pozzolanic SCM particles, such as mechanical properties, particle size distribution, and reactivity noticeably influenced the produced concrete. The natural pozzolan acted as reinforcing particles, leading to the formation of hydration products.

Celik et al.^5^ investigated the effects of high-volume SCM on the strength and durability of self-consolidating concrete. Natural pozzolan and fly ash were employed as the high volume SCMs. It was concluded that both types of SCMs performed satisfactorily in the mixtures. The main properties of eco-friendly concrete were like those of the specimens made from sole OPC. In another study by Uzal and Turanli^6^ volcanic tuffs as SCM were incorporated in the OPC blends. Blended cement with 55% of SCM showed similar *CS* development as the reference cement based on sole OPC. Najimi et al.^7^ use up to 30% of natural zeolite in OPC concrete. The use of blended cement with this type of SCM led to positive development of concrete durability such as chloride ion penetration, but the durability in acid environmental was low.

With high volumes of SCM, the durability properties of concrete usually get improved. There are several studies on OPC substitution, and these concretes with high volumes of SCM have better durability properties compared to sole OPC concretes. Hamada et al.^8^ stated that the properties of SCM are close relayed to the durability of concrete. The fineness of oil fuel ash as SCM positively influenced on the durability of concrete in the acidic environmental. Another paper by Nguyen et al.^9^ investigates the impact of natural pozzolan on main properties of concrete immersed in seawater. The incorporation of natural pozzolan up to 15% led to positive impact on the durability in seawater environmental. The long-term strength of concrete was higher for the samples with natural pozzolan compared to the reference samples based on OPC concrete. Cheng et al.^10^ incorporated ground blast furnace slag to improve the durability of concrete systems in the marine environmental. Samples based on OPC deteriorated due to influence of chloride and sulphate compounds during drying-wetting cycles. The cracks formed because of the formation of secondary ettringite. The additive of ground blast furnace slag led to an improvement of the resistance to corrosion in sea environmental causing the formation of additional amounts of C–S–H gel that compacted the microstructure of the systems. Moreover, eco-friendly concrete mixtures with natural zeolite were investigated by Shahmansouri et al.^11^. The incorporation of natural zeolite up to 20% led to increase the strength and durability properties of concrete. In summary, it can be stated that the application of high-volume SCM is an efficacious way to improve the long-term properties of concrete.

### Usage of zeolitic SCMs

One of the possible pozzolanic SCMs is zeolite, which is conformed of microporous aluminosilicate particles. The role of zeolite on the cement hydration was studied by Vejmelková et al.^12^, who included a natural zeolite as the SCM (up to 60%). The study concluded that the optimal composition of the binding material resulted when 20 wt.% of OPC was substituted by the natural zeolite, since the main properties of the specimens were like those exhibited by the reference specimens (composed entirely of OPC). In this case, some of the important durability properties of the specimens were improved, such as chemical resistance and frost resistance. Kocak et al.^13^ conducted a study related to blended cements with a natural zeolite based in clinoptilolite mineral. The results of the experiments showed that clinoptilolite, depending on its amount, remarkably affected the main properties of the blended cement. This effect was found to be related to the fact that zeolite chemically binds the Ca(OH)_2_ compounds, which are formed during the hydration of OPC, so forming compounds similar to the ones propter of cement hydration. However, since zeolite is a pozzolanic material, these reactions of blended binders are usually much slower than those of OPC hydration, and only after 180 days of hydration strength properties exceeds the strength values of OPC concrete^14^. Uzal et al.^15^ incorporated 55 wt.% clinoptilolite in the blended cements. After 28 days of hydration the mortar prepared with this blended cement had similar strength properties to those of OPC mortar strength. In the mineral composition of hardened cement paste free Ca(OH)_2_ was not detected, the amount of large pores (more than 50 nm) decreased and clinoptilolite converted to hydration products after 28 days.

Hence, the usage of high-volume zeolite as SCM to improve the durability properties of concrete has been already investigated by various authors.

### Usage of additives to accelerate the pozzolanic reaction

Since pozzolanic reactions are slow, different methods to speed up them are usually employed. One of the solutions to accelerate the reactions could be the use of chemical additives. Shi et al.^16^ investigated the blends made from natural pozzolan 80% and hydrated lime 20%. Various additives (Na_2_SO_4_, CaCl_2_·2H_2_O and NaCl) were incorporated in this system. The early strength of samples was significantly improved by using 4% Na_2_SO_4_ additive. The delayed strength values improved with the CaCl_2_·2H_2_O additive. On the other hand, the usage of two different additives such as CaCl_2_·2H_2_O + Na_2_SO_4_ or Na_2_SO_4_ + NaCl·CaSO_4_·0.5H_2_O did not affect positively the strength of the specimens.

An alternative way to increase the rate of the pozzolanic reactions is the incorporation of hydrated lime (Ca(OH)_2_) or quicklime (CaO) to the cementitious system. Al-Amoudi et al.^17^ investigated OPC concrete containing a blend of natural pozzolan and hydrated lime as the SCM. This SCM led to the improve of early and delayed strengths. From the durability point of view, these specimens performed in a much more satisfactory way than the specimens containing OPC and natural pozzolan, but without hydrated lime. Zhou et al.^18^ used sea water for the acceleration of pozzolanic reactions. Sea water was used for the preparation of sewage sludge ash and lime blends as binders. The influence of two main sea salts such as NaCl and MgCl_2_ on the blend cement systems was studied. These salts led to the decrease of the pore size in the samples and, at the same time, to a higher amount of small pores. Besides, Friedel’s salt formed in the blends with NaCl, accelerating the hydration process and increasing the early strength. MgCl_2_ addition also accelerated the hydration due to the formation of Mg(OH)_2_ and improved the later (60 days) strength of the systems. In a study by Mei et al.^19^, a double component additive made from sodium sulphate and nano-SiO_2_ was investigated for the accelerate fly ash steam-cured cement systems. The synergetic effect of these components was determined, and the additive improved the strength properties and the hydration rate. Hence, it was concluded that it is possible to incorporate high-volume fly ash in the cement systems, although its usage is limited without this double additive.

From the performed review, it can be concluded that the usage of pozzolanic high-volume SCMs is an effective way to reduce the OPC content in concrete, causing a significantly positive impact to climate change and, when industrial wastes are employed, contributing to circular economy. However, the usage of pozzolans instead of OPC brings some important drawbacks, such as the decrease of the mechanical performance and the slowing of the hydration reaction. Therefore, the aim of the current study consists in investigating the durability properties of OPC mortars containing high-volume zeolite, but correcting the mentioned drawbacks by including pertinent additives: quicklime (to foster the formation of calcium hydration products), and mineral accelerators (to speed the pozzolanic reaction). The synergetic effect of the zeolitic SCM, the quicklime, and the mineral accelerators on the mechanical and durability properties of the specimens is investigated and discussed.

## Experimental materials and methods

### Initial materials

The employed binding material was OPC of CEM I 52.5R category. The OPC was partially substituted in some specimens by a zeolitic SCM. Two types of zeolites were alternatively employed: a natural zeolite (NZ) and synthetic waste one (FCC). The latter is a waste material from the petrochemical industry, where it was used as a catalyst in the fluid catalytic cracking process, eventually getting polluted and becoming a waste material.

**Figure 1:**
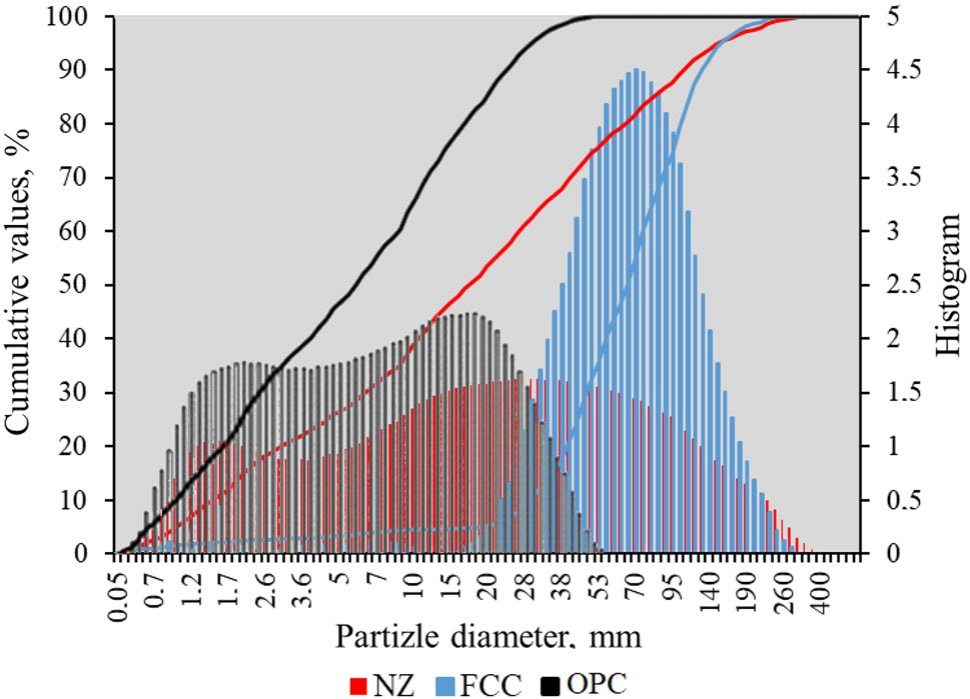
Particle size distribution of the initial materials: NZ, FCC and OPC.

The oxide composition of the initial materials is given in Table 1, where the differences among the zeolites can be easily noticed. NZ is remarkably richer in silicon oxide than FCC, whereas the aluminium oxide content presents the opposite tendency. Similar oxide composition of FCC and NZ was determined by Yu et al.^20^ and Kitsopoulos^21^, respectively. Moreover, the particle size distribution of the materials is provided in Fig. 1. It can be observed that the FCC particles are relatively coarse, with an average diameter (∅_*m*_) of 80.99 μm, and a narrow scatter. The NZ particles are smaller (∅_*m*_ of 42.38 μm), with a much wider scatter. The OPC particles are the smallest ones (∅_*m*_ of 5.19 μm), also with a wide scatter.

**Table 1:** Oxide composition of the initial materials, identified by XRF analysis.

Material	SiO_2_	CaO	Al_2_O_3_	Fe_2_O_3_	MgO	K_2_O	Na_2_O	SO_3_	Cl^-^	TiO_2_	Other
OPC	21.22	66.36	3.84	2.92	2.31	0.23	0.18	0.25	–	0.29	2.4
NZ	72.57	3.29	12.45	1.72	0.63	3.59	0.24	–	–	–	5.51
FCC	35.34	0.36	48.78	1.24	0.72	0.06	0.36	0.35	2.56	3.54	6.69

**Figure 2:**
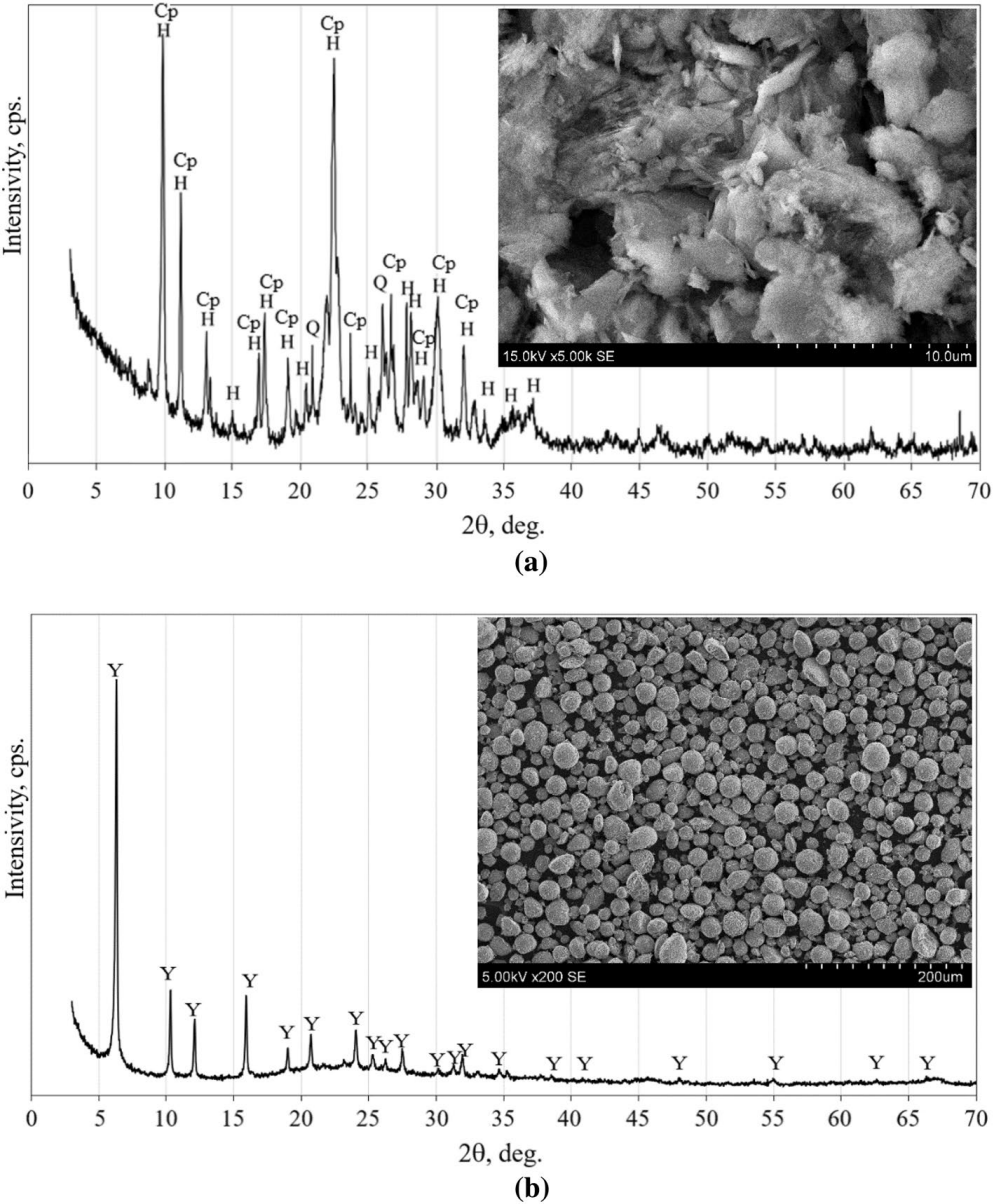
XRD mineral composition of NZ (**a**) and FCC (**b**). Notes: Y – aluminium silicate hydrate Al_60.352_∙Si_139_∙O_371.52_∙H_5.984_ (PDF No 73–2313); Cp – clinoptilolite (Na,K,Ca)_6_(Si,Al)_36_O_72_∙20H_2_O (25–1349); H – heulandite CaAl_2_Si_7_O_18_∙6H_2_O (19–212); Q – quartz SiO_2_ (83–2465).

The mineral composition of the NZ and FCC was identified from the XRD patterns given in Fig. 2a and b, respectively. According to the observed XRD patterns, NZ is based in clinoptilolite and heulandite minerals with small quantities of quartz. By using XRD analysis it is difficult to distinguish the phases of heulandite and clinoptilolite due to structural similarity. In FCC predominates aluminium silicate hydrate which is of faujasite type.

The microstructure of both types of zeolites is shown in the Fig. 2a and b. Clinoptilolite is characterized by a layered structure with a regular lamellar conglomerate^22^. Different microstructure had FCC. In this case the round shape particles dominated^23^.

The Chapelle test revealed a 2.13 times higher FCC pozzolanic activity (1.157 g/g, expressed as the ratio between masses of the reacted Ca(OH)_2_ and the pozzolan), than that of the NZ (0.543 g/g).

### The preparation of initial materials mixtures

The mixture was prepared by mixing 25 wt.% binding material with 75 wt.% standard sand. The employed water/binder (W/B) ratio was 0.5 for reference mixture (1). By substituting OPC with SCM water/binder increased due to porous structure of zeolites. Mixture composition and water/binder ratio were varied in order to maintain the same workability of the mixes in S2 class according to the slump test. The binding material was composed by 50 wt.% OPC, 40–50 wt.% of SCM (either FCC or NZ) and 0–10 wt.% of quicklime (CaO). The quicklime is included as an additional calcium source for the pozzolan to evolve into C-S-H. Additionally, in some cases 4 wt.% of inorganic accelerator was added (either NaCl or Na_2_SO_4_), calculated from the binding material. The binding material of the reference specimen consisted exclusively in OPC. The components of the various binding materials are Table 2. The naming of the various binding materials can be explained as follows: the numbering indicates the type of SCM (1—reference specimen without SCM; 2—FCC, 3—both FCC and NZ; 4—NZ); “S” and “C” indicate the presence of 4 wt.% accelerator (either Na_2_SO_4_ or NaCl, respectively); finally, “L” means that the specimen contains 10 wt.% of CaO, whereas the absence of “L” indicates that CaO is not included.

**Table 2:** Compositions of the binding materials.

Group	Mix name	OPC, wt.%	FCC, wt.%	NZ, wt.%	CaO	Na_2_SO_4_, %	NaCl, %	W/B ratio
No accelerators	1	100	–	–	–	–	–	0.50
	2	50	50	–	–	–	–	0.63
	3	50	25	25	–	–	–	0.61
	4	50	–	50	–	–	–	0.60
	2L	50	40	–	10	–	–	0.63
	3L	50	20	20	10	–	–	0.62
	4L	50	–	40	10	–	–	0.61
With Na_2_SO_4_	1S	100	–	–	–	4	–	0.50
	2S	50	50	–	–	4	–	0.62
	3S	50	25	25	–	4	–	0.60
	4S	50	–	50	–	4	–	0.60
	2SL	50	40	–	10	4	–	0.63
	3SL	50	20	20	10	4	–	0.61
	4SL	50	–	40	10	4	–	0.61
With NaCl	1C	100	–	–	–	–	4	0.50
	2C	50	50	–	–	–	4	0.62
	3C	50	25	25	–	–	4	0.61
	4C	50	–	50	–	–	4	0.60
	2CL	50	40	–	10	–	4	0.63
	3CL	50	20	20	10	–	4	0.61
	4CL	50	–	40	10	–	4	0.62

The pouring, compacting, and curing processed of the mortar specimens were performed according to the indications provided in standard EN 12390-2^24^. This standard describes the requirements for the preparation and filling of the moulds, for the compaction of the mixture (in the current study, by using a vibrating table), for the surface levelling and for the curing of the specimens. The curing was performed (in accordance with the standard) in a chamber with conditions of 20 ± 2 °C temperature and > 95% relative humidity.

### Experimental techniques

The visual study of the microstructure of the specimens was performed through the Scanning Electron microscope *Hitachi S*-3400*N Type II*. Parameters: image resolution of ≥ 3 nm or ≥ 10 nm (with Δ*V* either 30 kV or 3 kV, respectively); acceleration voltages of 10 kV for the performed enhancement.

The element composition of the initial materials was depicted via X-ray fluorescence analysis, by using the *Bruker X-ray S*8 *Tiger WD* spectrometer, with a rhodium tube. Parameters: anode voltage of ≤ 60 kV; electric current of 160 mA; helium atmosphere to process the powder samples; the SPECTRA Plus QUANT EXPRESS method was employed to depict the results.

The X-ray diffractometer *D8 Advance* (Bruker AXS, Karlsruhe, Germany) was employed to determine the mineral composition of the initial materials and the created specimens. Parameters: CuKα radiation Ni filtered; 40 kV tube voltage, 40 mA tube current. The diffraction patterns were recorded in the Bragg–Brentano geometry within the 2θ range of 3°–70° at a scanning speed of 6° min^−1^. The XRD patterns were assigned to specific minerals from the PDF-2 database by using *Oxford Cryosystems Crystallographica Search-Match* software.

The laser particle size analyser *Cilas* 1090 was employed to study the particle size distribution of the initial materials. Parameters: dispersion mode; dispersion medium – air; measuring rates between 0.1 μm and 500 μm.

The pozzolanic activity of both zeolites types (FCC and NZ) was determined via Chapelle test^25^. This test determines the binding of Ca(OH)_2_ in zeolite in an aqueous suspension: 1 g of zeolites reacts with 1 g Ca(OH)_2_ in 200 mL of water at 90 °C for 16 h.

The suitable consistence of the fresh concrete mixture was determined according to the slump test procedures described in standard LST EN 12350-2^26^. Thereby, a satisfactory mixing water content could be selected.

The density of the hardened concrete specimens was determined according to the standard EN 12390-7^27^. The volumes of the specimens were calculated by measuring their actual measurements. The masses were determined for oven-dried specimens, heated at a temperature of 105 ± 5 °C. The drying process is considered to be finished when the mass changes by less than 0.2% in a 24 h period.

The determination of the *CS* of the concrete specimens (after 28 days of curing) was carried out with the computerised press *ToniTechnik* 2020.0600/132/02, according to the requirements given European standard EN 12390-3^28^, which determines the main conditions and requirements of the test: loading rate of 0.6 ± 0.2 MPa/s and room temperature of 20 ± 5 °C. The specimens should be tested within 10 h from its removal from the curing. The measuring rate of the press was set to be 0.02 s.

Moreover, the flexural strength (*FS*) of the concrete specimens (after 28 days of curing) was tested according to the standard EN 12390-5, Annex A^29^, by applying a centre-point load to 40 × 40 × 160 mm prismatic specimens. The loading rate was 0.05 MPa/s.

The total and open porosity of the concrete specimens (after 28 days of curing) was evaluated by water absorption according to the methodology indicated by Girskas and Skripkiunas^30^. Meanwhile, the water absorption of the specimens was determined according to standard GOST 12730.4-78.

The freeze–thaw resistance was evaluated according to Lithuanian standard LST 1428-17^31^, according to which the concrete specimens were submerged in a 18 ± 5 °C water bath and then stored in a − 18 ± 2 °C freezer. This cycle was performed a certain number of times (till visual damage is depicted), and mass and strength losses were determined.

The carbonisation of the concrete specimens (after 28 days of hydration) was carried out in a FDM environmental chamber (F.IIi Della Marca), according to standard EN 14630^32^. For 78 days, the specimens were kept under conditions of 20 °C temperature, 5% CO_2_ concentration and 95% relative humidity. Eventually, the phenolphthalein test was performed. The specimens were fractured in two parts, and the fracture surface was sprayed with phenolphthalein. The presence of unreacted portlandite (Ca(OH)_2_) is revealed by the violet colour gained by the surface. If the surface does not turn violet, it means that portlandite has carbonized into calcite (CaCO_3_).

## Results and discussion

The effect of the zeolitic CSM, quicklime and inorganic accelerators on the properties of the various mortars have been investigated:

1. Physical–mechanical properties: density, *CS* and *FS* after 28-day curing.
2. Durability properties: water absorption, porosity, freeze–thaw resistance, resistance to carbonation-induced corrosion.
3. Exposure to sea water: change of mass and of *CS*, mineral composition.

The main results are provided and discussed in the following subsections.

### Physical–mechanical properties

**Figure 3:**
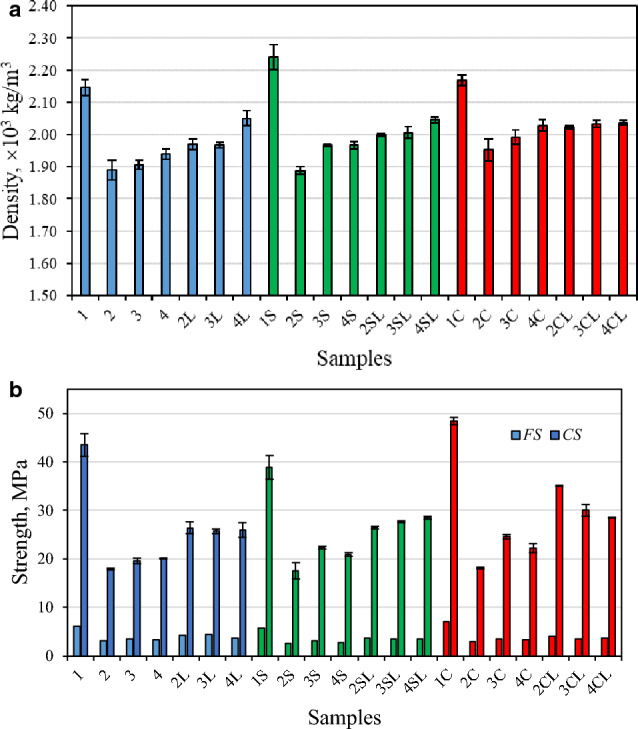
Density (**a**) and strength (**b**) tendencies of the specimens after 28 days of curing.

The density values of the hardened dry specimens (after 28 curing days) are provided in Fig. 3a, whereas the *CS* and *FS* are provided in Fig. 3b. The behaviour of reference specimens (1, 1S and 1C, without zeolitic SCM or quicklime) reveals that the addition of the Na_2_SO_4_ accelerator (specimen 1S) caused a noticeable increase of the density values. On the other hand, the increase in density caused by NaCl accelerator (specimen 1C) was not significant. However, the mechanical strength results do not correlate with the density, since the Na_2_SO_4_ reduced the *CS* by about 11%, while the NaCl accelerator exhibited the opposite tendency: it enhanced the *CS* by 11%, in respect to the specimen without accelerators. The same trend is followed by *FS* results.

From Fig. 3, it can be observed that the addition of zeolites as high-volume SCM in all systems caused a significant impact on the density of the specimens (reducing it by 8–15%) and, subsequently, an abrupt decrease of their mechanical strength (by 49–63%). As explained by Zheng et al.^33^, this may be determined by the insufficient amount of hydration products: C-S-H and Ca(OH)_2_. Hence, it can be stated that the substitution of OPC by high amounts of zeolite does not compensate the OPC in the cementitious systems, at least from the point of view of the mechanical properties. However, when quicklime was included (as an additional source of calcium), the negative impact was palliated: the average density of the specimens grew up to 10% and the strength correspondingly increased even by 40%, in respect to the respective specimens without quicklime (although remaining lower than the strength of the reference specimens, which do not contain zeolitic SCM). This effect is explained by Yılmaz et al.^34^, who determined that the strength of OPC systems with partial zeolite replacement depends on the amount of Ca(OH)_2_ formed in the system, which, reacting with zeolite, forms hydration products that positively affect the strength of concrete.

Considering the joint effect of all components (zeolitic SCM, quicklime and accelerators), it can be noticed that the introduction of the Na_2_SO_4_ accelerator (see Fig. 3 results marked in green) did not make a significant impact on the strength indicators of the specimens containing zeolitic SCM (in respect to the respective specimens without accelerator), while the presence of NaCl salt (see columns marked in red) remarkably enhanced the *CS* values of the specimens (especially when quicklime is included), reaching for specimen 2CL the maximum value of 35.1 MPa, i.e. almost two times higher than that of the respective specimen without quicklime and accelerator (specimen “2”, *CS* = 18.0 MPa) and 1.3 times higher than that the respective specimen with quicklime but without accelerator (specimen “2L”, *CS* = 26.4 MPa). Moreover, the introduction of NaCl leads to a decrease in the pore size, which has a positive effect on the strength characteristics^18^.

In general terms, the results given in Fig. 3 reveal that the addition of quicklime can compensate the loss in both strength and average density values caused by the addition of zeolitic SCM.

### Durability properties

The crystal lattices formed in OPC concrete are frequently exposed to water and salt solutions and to various weather conditions. The effect of these aggressive factors on the dense crystal matrix of concrete is rather slow, so that their short-term influence is neither noticeable nor significant. However, in the long-term, these external aggressive conditions cause important deterioration processes, in the same way as happens with the other construction materials. In this context, the main positive effect of pozzolan SCM consists in improving the durability properties of concrete, since their lately formed hydration products close the pores within the crystal matrix of the specimens. Of course, the positive or negative effect of SCM on the main properties of the blends strongly depends on the replacement percentage and the type of SCM^1,13^.

Therefore, in this section, the durability of the various specimens was evaluated by considering several parameters: water absorption, porosity, freeze–thaw resistance, and carbonation resistance.

#### Water absorption, porosity, and freeze–thaw cycles

**Figure 4:**
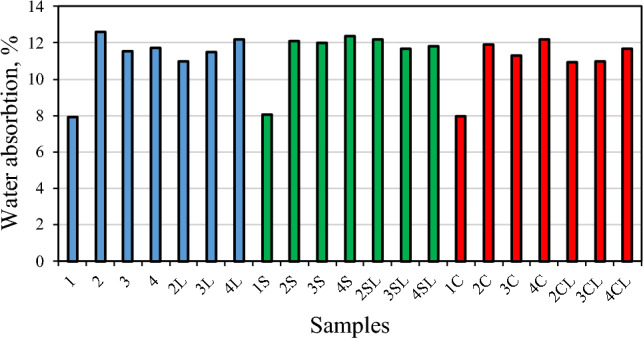
Water absorption of the specimens after 28 days of curing.

The water absorption results of each specimen are given in Fig. 4. It can be observed that the water absorption of the reference specimens (1, 1S and 1C) is approximately 8%, a remarkably lower value than those exhibited by the specimens with zeolitic SCM (about 12%). This behaviour is related to the porous nature of the zeolite particles, which enhance the absorption of water within them. On the other hand, the influence of the quicklime (specimens marked with “L”) on water absorption is, in the majority of the cases, positive: the values of water absorption slightly decrease or remain the same. In the specimens with high-volume zeolitic supplementary cementitious materials the amount of free Ca(OH)_2_ was reduced due to smaller amount of OPC. When CaO is included in the system, the zeolite gets the possibility to react with lime, so forming an additional amount of hydration products which densified the microstructure and consequently reduced its water absorption. The chloride additive though (marked in red colour), slightly reduces the water absorption of the specimens in respect to that of the specimens with sulphate additive (marked in green colour).

**Figure 5:**
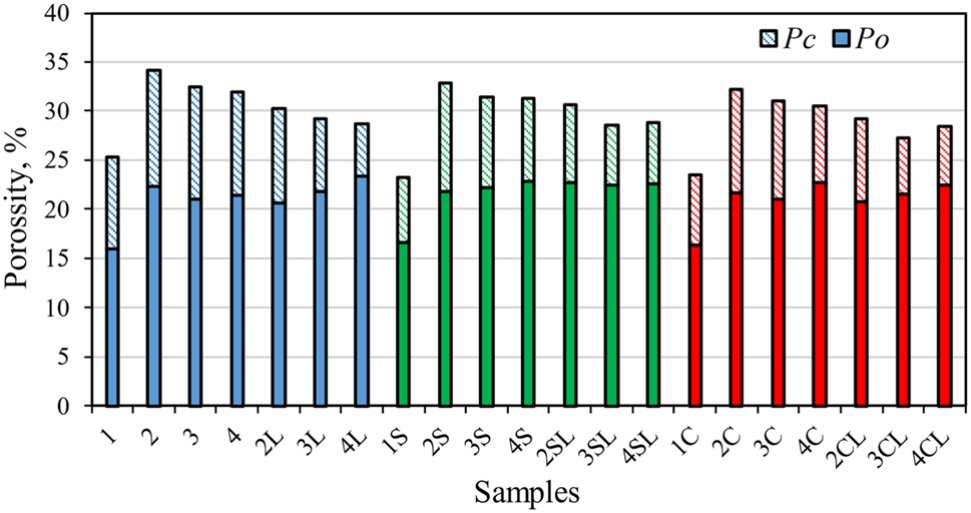
Open porosity (P_o_) and close porosity (P_c_) of the specimens after 28 days of curing.

Moreover, durability is investigated from the point of view of porosity (see Fig. 5). The tendencies observed for the open porosity (*P*_*o*_) tightly correlate to the water absorption, i.e., the reference specimens present lower *P*_*o*_ levels, and the influences of the other factors (type of zeolite, type of accelerator and quicklime content) are rather insignificant. However, if the close pores (*P*_*c*_) are also considered, the overall porosity values (*P*_*o*_ + *P*_*c*_) show interesting tendencies. Of course, the reference specimens (1, 1S and 1C) exhibited a lower total porosity than the rest ones, which contain zeolite. However, if only the specimens with zeolitic SCMs are considered, it can be observed that the main *P*_*c*_ influencing factor is the presence of quicklime. When quicklime is included in the mixture (specimens with “L”), the *P*_*c*_ values gets significantly reduced, causing a total porosity (*P*_*o*_ + *P*_*c*_) decrease of 7%-12%. This effect is even higher in the specimens with both FCC and NZ (specimens marked with “3”) and with only NZ (marked with “4”) than in those containing only FCC (marked with “2”) as the SCM. As already mentioned, this porosity reduction seems to be related to the formation of additional hydration compounds (such as C-S-H and calcite) when the quicklime is included in the mixture. Moreover, the usage of NaCl and NaSO_4_ accelerators slightly reduced the porosity in respect to the specimens without accelerators, by 2–8%. This behaviour has been also depicted by various authors in similar investigations. H. Nguyen^35^ stated that for high volume fly ash concrete Na_2_SO_4_ shortened the setting time of the mixture and improved the early mechanical strength of the specimens. The improvement was found to be related with the better dissolution of the pozzolans (in this case, the fly ash) when the sulphate additives were included. Pang et al.^36^ determined that the addition of NaCl increased hydration rate of blended cement systems. Hence, it can be concluded that the addition of quicklime and of inorganic accelerators reduced the porosity of the specimens containing zeolitic SCM.

**Figure 6:**
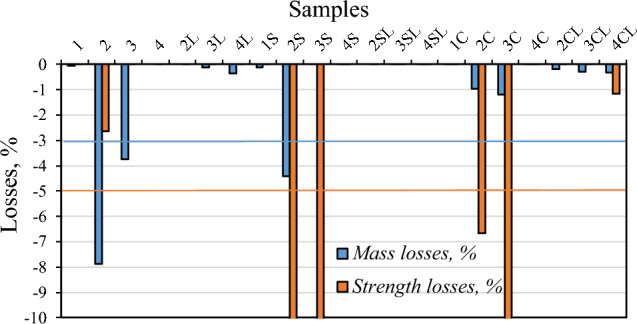
Mass losses and strength losses of the specimens after 28 days of curing and the freeze–thaw resistance test (46 cycles).

The effect of freeze–thaw cycles is an important component of the evaluation of the durability of a building material. Figure 6 gives the mass and *CS* losses after the freeze–thaw cycles. It can be determined that the specimens including NZ (alone or combined with FCC) as SCM did not satisfy the requirements of either ≤ 3% or ≤ 5% for the mass or strength loss, respectively, raised by standard LST 1428-17^31^. On the other hand, the specimens including FCC performed adequately. When quicklime was included, all the specimens passed the durability test. The positive effect of quicklime content to the freeze–thaw resistance of the specimens seems to be related to their porosity reduction, due to the formation of additional hydration compounds (C-S-H and CaCO_3_).

Some explanations to these observed results are provided by various authors. According to T. Milović et al.^37^, the use of NZ up to 10% could have a positive effect of the freeze–thaw performance in blended cement. This improvement is closely related with the decrease of the *P*_*o*_ in the hardened specimens. On the other hand, it was determined that the incorporation of a larger amount of NZ (higher than 20%) in the system provoked a negative effect on the frost resistance. In another study by Markiv et al.^14^ the increase of resistance to freezing and thawing damage of concrete with 10% of NZ is explained by the formation of secondary C-S-H gel. In this case the capillary porosity was decreased due to the formation of additional amount of hydration compounds. So, more than 10% incorporating of pozzolan (zeolite) led to decrease of freezing and thawing resistance of concrete. In the current study the including of high-volume zeolitic supplementary cementitious materials had negative effect on the frost resistance but the use of quick lime improved this freezing and thawing resistance of concrete.

#### Carbonation resistance

Carbonation-induced corrosion occurs when hardened concrete is exposed to water containing dissolved CO_2_ (carbonic acid)^38,39^. The reaction between calcium hydroxide and CO_2_ is developed in two steps as explained by numerous authors^40–42^. Firstly, calcite (CaCO_3_) precipitates when portlandite reacts with the carbonic acid (see Eq. (1)). Subsequently, calcium carbonate hydrate appears due to the participation of water and CO_2_ (see Eq. (2)).

1 $$ {\text{Ca }}\left( {{\text{OH}}} \right)_{{2}} + {\text{ CO}}_{{2}} \to {\text{ CaCO}}_{{3}} + {\text{ H}}_{{2}} {\text{O}}, $$

2 $$ {\text{CaCO}}_{{3}} + {\text{ CO}}_{{2}} + {\text{ H}}_{{2}} {\text{O }} \leftrightarrow {\text{ Ca}}\left( {{\text{HCO}}_{{3}} } \right)_{{2}} , $$

**Figure 7:**
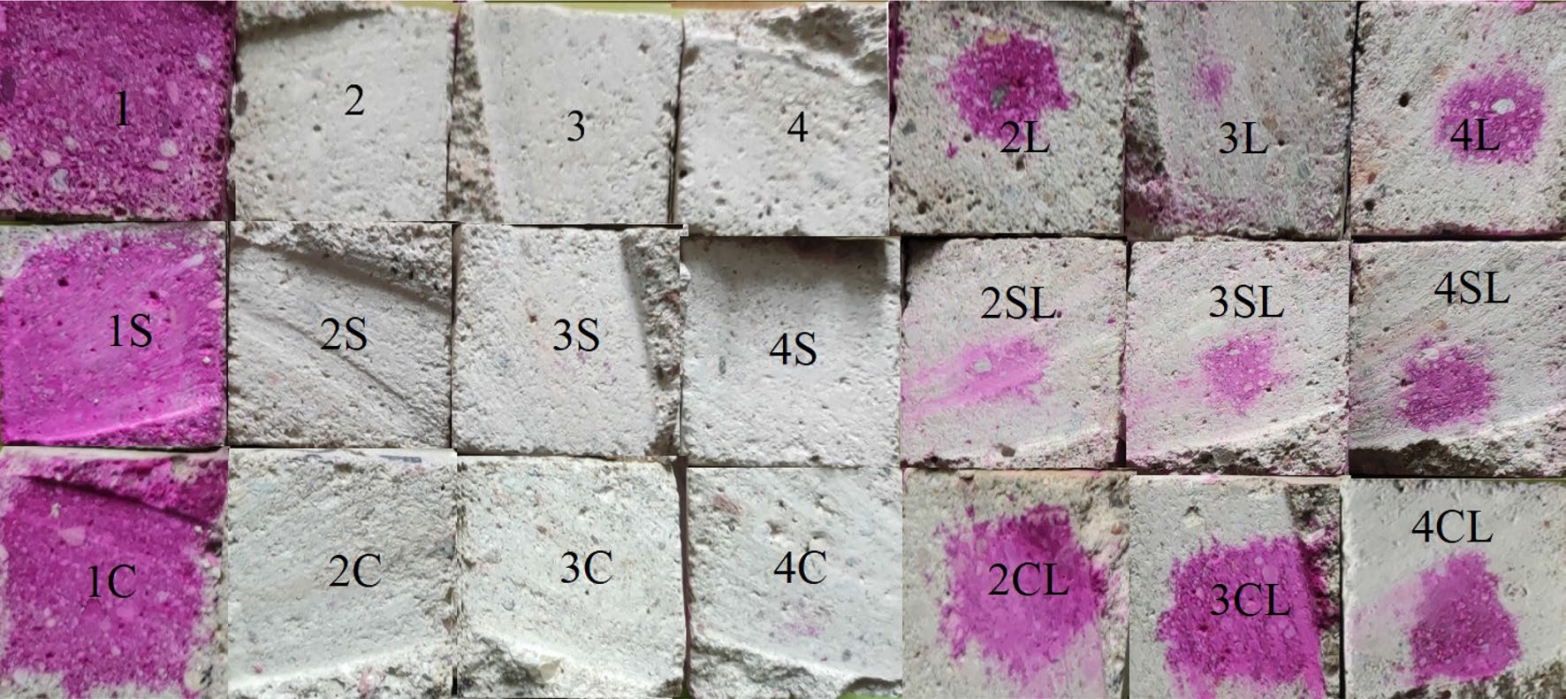
Results of the carbonation test after the application of phenolphthalein (violet colour indicates the presence of Ca(OH)_2_.

The durability of concrete depends on its composition, i.e. on the type and quality of its components. The images of the specimens after the carbonation test are given in Fig. 7. From the images of Fig. 7 it can be noticed that the specimens with FCC and NZ zeolites and without quicklime (2–4; 2S-4S; 2C-4C) did not contain any portlandite. This result coincide with the already commented fact that quicklime additive reduces the porosity of the specimens (see Fig. 5) and improves their resistance to freeze–thaw cycles (see Fig. 6), confirming that remaining portlandite reacts with the zeolitic SCM forming C-S-H compounds, so that the Ca(OH)_2_ gets exhausted. This causes an improvement of the crystal lattice of the specimens, but reduces the carbonation resistance, since portlandite is necessary to stabilise the concrete against carbonate corrosion, inside the specimens. When 10 wt.% quicklime was added to the mixtures (2L-4L; 2SL-4SL; 2CL-4CL), the carbonation resistance got improved, since the depth of carbonation varied from 8 to 18.75 mm, with portlandite still present in the internal zone of the specimen, not affected by carbonation. However, the reference samples (1, 1S, 1C) exhibited the lowest carbonation level, with carbonation depths ranging from 0 to 2.75 mm. This was caused by the lower porosity, lower water absorption and the presence of portlandite in these reference specimens.

Moreover, it was determined that carbonation caused a mass enhancement of the specimens from 1.8 to 3.06%. It can be attributed to a heterogenic reaction of CO_2_ and the portlandite included in the specimens (see Eq. (1)). Since the XRD patterns did not depict the presence of Ca(HCO_3_)_2_, it can be stated that the reaction Eq. (2) did not take place in the applied conditions. The mass increase and carbonation depth values are given in Table 3.

**Table 3:** Carbonation depth and carbonation-induced mass increase of the specimens.

Specimen	Carbonation depth, mm	Increase of mass, %	Specimen	Carbonation depth, mm	Increase of mass, %
1	0	2.44	2SL	15.00	2.48
2	Fully carbonated	2.49	3SL	16.50	2.44
3	Fully carbonated	1.80	4SL	16.00	2.56
4	Fully carbonated	2.21	1C	2.75	1.86
2L	13.25	3.06	2C	Fully carbonated	2.74
3L	18.75	2.72	3C	Fully carbonated	2.69
4L	16.25	2.64	4C	Fully carbonated	2.20
1S	0.50	1.99	2CL	8.75	2.11
2S	Fully carbonated	1.60	3CL	8.00	2.04
3S	Fully carbonated	1.96	4CL	13.75	2.84
4S	Fully carbonated	2.13			

Hence, the durability properties seem to be mostly influenced by the presence of quicklime in the system, which encouraged the formation of additional hydration products, such as C-S-H and calcite.

### Durability properties in sea water (SW)

This section discusses the durability of the produced specimens when exposed to aggressive sea water conditions. To evaluate the effect this kind of exposure, the specimens where immersed for 28 days in either sea water (SW) or distilled water (DW). The durability properties of the specimens exposed to these alternative conditions are further provided and discussed.

**Figure 8:**
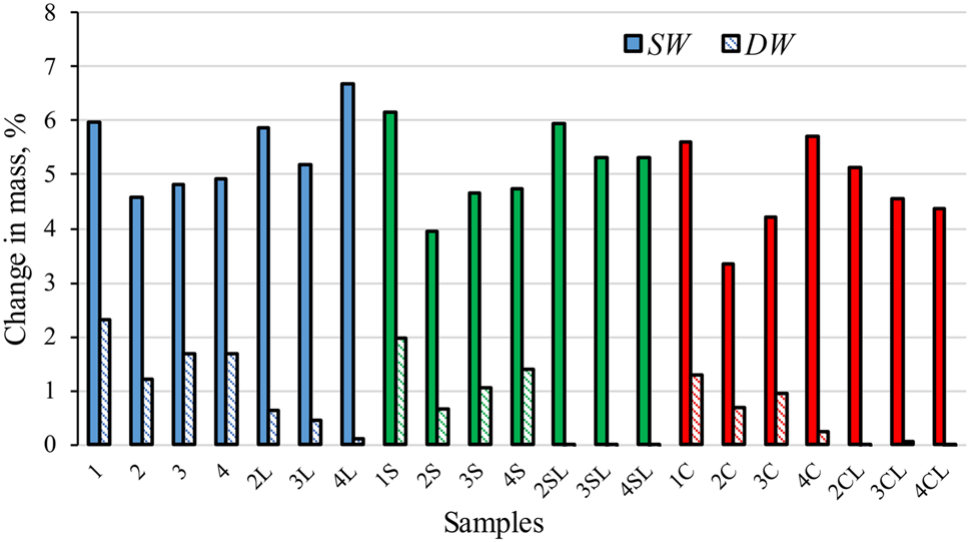
Mass changes of the dry specimens after 28 days immersion in sea water (SW) or distilled water (DW).

**Figure 9:**
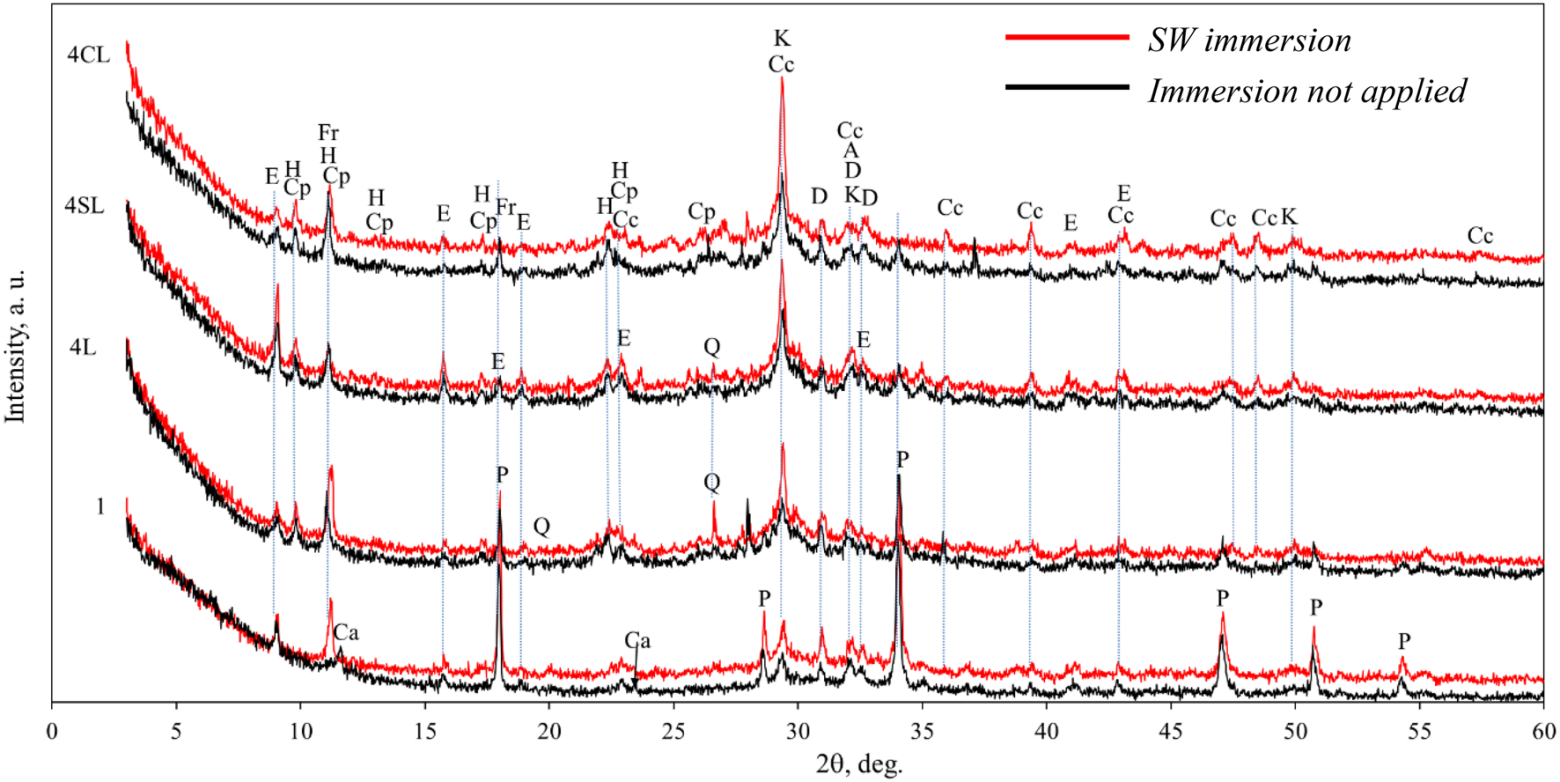
X-ray diffraction patterns of representative specimens after SW immersion (red) and with no immersion applied (black). Notes: K – calcium silicate hydrate Ca_1.5_SiO_3.5_∙xH_2_O (PDF No 33-306); Cp – clinoptilolite (Na,K,Ca)_6_(Si,Al)_36_O_72_∙20H_2_O (25-1349); H – heulandite CaAl_2_Si_7_O_18_∙6H_2_O (19-212); Cc – calcium carbonate CaCO_3_ (72-1937); P – calcium hydroxide Ca(OH)_2_ (84-1265); Fr – Friedel’s salt Ca_2_Al(OH)_6_Cl·2H_2_O (78-1219); E – Ettringite 6CaO·Al_2_O_3_·3SO_3_·32H_2_O (72-646); A – Alite 54CaO·16SiO_2_·Al_2_O_3_·MgO (13-272); D – Larnite 2CaO·SiO_2_ (83-461); Q – Quartz SiO_2_ (83-2465); Ca – Calcium aluminium oxide carbonate hydrate Ca_4_Al_2_O_6_ CO_3_ 11H_2_O (No 41-219).

The increase of mass (Δ*m*) of the dry specimens after immersion (in respect to the mass of the specimens before immersion) is provided in Fig. 8. The Δ*m* values of specimens after exposure to SW were within the range 3.34–6.68%, much higher in all cases than the Δ*m* of the respective specimens exposed to DW (from − 0.09 to 2.31%). This difference may be explained the fact that the DW caused a Δ*m* in the specimens by improving the OPC hydration, while the SW additionally increased the Δ*m* by forming additional new compounds, such as Friedel’s salt or ettringite, resulting from the contact of the specimen surfaces with the chloride and sulphate ions contained in SW. The formation of these compounds is revealed by XRD analysis of representative specimens given in Fig. 9.

When no quicklime was included, the reference specimens (1, 1S and 1C) exhibited the highest Δ*m* of their respective groups in both DW and SW. The effect of quicklime on the Δ*m* of the specimens was found to be strongly related to the type of water they were immersed in. When specimens were exposed to DW, the Δ*m* of the specimens including quicklime was much lower than those of the specimens not containing it. However, when immersion in SW was applied, the opposite tendency was observed, even surpassing the Δ*m* of the reference specimens. The reason of this tendency seems to be related to the fact that the quicklime enhanced the Ca(OH)_2_ content of the specimens, so fostering an even higher formation of the new compounds within them.

The XRD mineral compositions of some representative specimens after immersion in SW (see Fig. 9) provides some light to understand more deeply the described Δ*m* tendencies. In general terms, it can be observed that the immersion in SW encouraged the formation of C-S-H phase. In the specimen with quicklime (4L, 4SL and 4CL) the soaking in SW led to the formation of additional amount of calcite. Meanwhile calcite almost was not depicted in the specimens not including quicklime. The presence of zeolitic SCM in the mixtures strongly reduced the portlandite compound, especially when accelerators were included. This fact seems to be related to the smaller amount OPC, which is substituted by zeolitic SCM in the mixtures. The highest C-S-H peaks are observed in the specimens including accelerators (4SL and 4CL), indicating the suitability of NaCl and Na_2_SO_4_ as reaction accelerators in the cementitious systems. The pattern of the reference specimen “1” shows that Friedel’s salt appears after immersion in SW, i.e. in a chloride bearing environment, as explained by various authors^43,44^.

**Figure 10:**
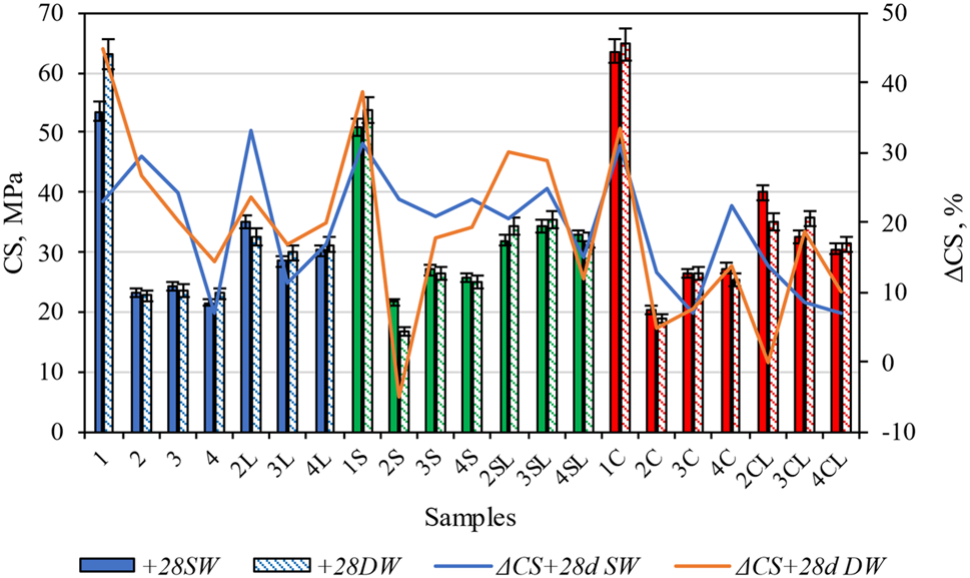
CS tendencies of the various specimens after 28 days immersion in SW (+ 28d SW) or DW (+ 28d DW) and their relative values (ΔCS) in respect to not-immersed specimens.

Figure 10 gives the *CS* values of the specimens after immersion and their percentual changes (Δ*CS*) in respect to the *CS* of the not immersed specimens (which were given in Fig. 3b). In most cases (except specimen 2S in DW) the *CS* values increase (Δ*CS* is positive). This behaviour was expected, since immersion encourages additional hydration of the OPC. The *CS* values of the control specimens (1, 1S and 1C) immersed in DW are in all cases higher than those of the respective specimens immersed in SW. Regarding to the specimens containing zeolitic SCM, it can be noticed that when quicklime is not included, they present higher *CS* values after immersion in SW than in DW, whereas the specimens containing quicklime do not exhibit clear *CS* tendencies in respect to the type of immersion water. Hence, the Δ*CS* tendencies do not necessarily correlate with the Δ*m* values observed in Fig. 8, where the mass of the specimens was observed to be clearly enhanced by immersion in SW and by their quicklime content. The Δ*CS* seems to be more related to the formation of new compounds (such as C-S–H and calcite) than to the sole compaction. B. Zhang, et al. published similar results similar results^43,44^. New hydration products densified structure of specimens which is close related with the mechanical properties. Furthermore, this study has showed the higher Δ*CS was determined for the samples with* quicklime and zeolites.

From the previous analyses it can be concluded that after immersion, the mechanical strength of the specimens increased, in both DW and SW. There is not a significant difference between the *CS* values of the specimens immersed in DW than those immersed in SW. Hence, it can be stated that when high-volume zeolitic SCM and quicklime are included in the mixtures, the exposure to SW environment does not damage the specimens, but even improves their mechanical performance. This positive impact is enhanced by the addition of inorganic accelerators.

**Figure 11:**
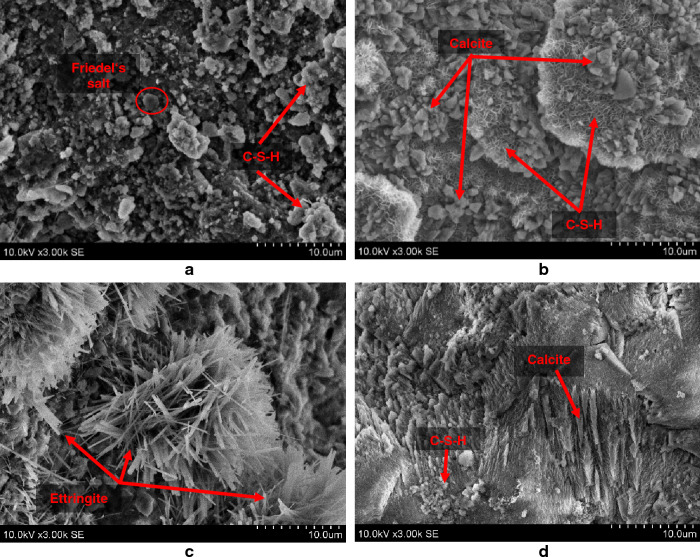
SEM images of representative specimens after SW immersion (× 3000): 1 (**a**), 4L (**b**), 4SL (**c**), 4CL (**d**).

The SEM microstructures of representative specimens with SW immersion are visualised in Fig. 11. These images confirm and complete the XRD mineral composition provided in Fig. 10. It can be seen that SW immersion provoked the formation of new compounds. In the reference specimen (marked as “1”) some particles with shapes of hexagonal plates and sizes of ~ 2 µm size were detected. According to these geometries, they seem to be Friedel's salt crystals. During immersion in SW ettringite reacted with chloride anions and Friedel's salt formed^33–35^. Moreover, in the same reference specimen, the phase C-S-H phase is also visible. On the other hand, the specimen 4L (with NZ and quicklime) presents a much different microstructure, with the predominance of flocculent C-S-H with inserted CaCO_3_ particles with prismatic shapes^35,36^. The specimens containing inorganic Na_2_SO_4_ and NaCl accelerators (4SL and 4CL, respectively) also presented specific microstructural features. In the 4SL specimen, mostly predominated needle-like prismatic ettringite crystals with flocculent and semi amorphous C-S-H phase. IN the 4CL specimen, the presence of NaCl led to the formation of a denser microstructure made from scaly calcite and semi amorphous C-S-H phases after soaking in water^37^. These observations confirm and complete the conclusions achieved through the XRD analysis given in Fig. 9.

The chloride (NaCl) could be the cause of corrosion of reinforcement in concrete. This corrosion risk could be determined and evaluated additionally in our next study. In this case we recommend do not use steel reinforcement in the concrete.

## Conclusions

During the current investigation, two types of zeolites (25–50%) were employed as high volume SCM in OPC mortars. Besides, quicklime (10%) was included as an additional calcium source, and NaCl or Na_2_SO_4_ (4%) as accelerators of the concrete hydration reaction. The influence of the various compositions on the mechanical properties and durability performance of the mortars were investigated, and the main conclusions are further described.

1. The mechanical properties of the specimens were severely reduced with the substitution of OPC with high-volume zeolitic SCM. However, the addition of quicklime palliated the situation, improving the *CS* values up to 40% in respect to those of the corresponding specimens not containing quicklime.
2. The quicklime addition to the specimens significantly improved the durability properties of the specimens, by reducing the close porosity, improving the freeze–thaw resistance and providing an additional portlandite source which stabilised the alkali environment against the damaging effect of carbonation. At the same time, the total porosity decreased by 7–12%, and with the addition of accelerators—by another 2–8%.
3. The addition of NaCl accelerator was found to be positive for the mechanical properties of the specimens, since it improved up to 1.3 times (specimen 2CL) the *CS* values of the specimens with quicklime, in respect to the specimen not containing accelerator. This positive effect is related to the formation higher amount of hydration products such as of C-S-H gel, Friedel’s salt and calcite.
4. The study of the durability of the specimens immersed in SW revealed the formation of new compounds such as Friedel’s salt and the increase of the amounts of ettringite, calcite and C-S–H. In this way, the exposure of the composite specimens to aggressive SW environment not only did not damage the specimens, but even enhanced their mechanical performance due to the formation of these new compounds.

Thus, the use of zeolite as a high-volume SCM brings both ecological and economic benefits, maintaining satisfactory mechanical properties and durability performance, especially when quicklime additive and NaCl inorganic accelerator are included.
